# Asymptomatic Esophageal Eosinophilia in an 11-Year-Old with Severe Persistent Asthma

**DOI:** 10.1155/2023/6678918

**Published:** 2023-10-12

**Authors:** Casey E. Hofstaedter, Runa Watkins, Nidhi Kotwal

**Affiliations:** ^1^Medical Scientist Training Program, University of Maryland School of Medicine, Baltimore, Maryland 21201, USA; ^2^Division of Gastroenterology and Hepatology, Department of Pediatrics, University of Maryland School of Medicine, Baltimore, Maryland 21201, USA; ^3^Division of Pulmonology and Allergy, Department of Pediatrics, University of Maryland School of Medicine, Baltimore, Maryland 21201, USA

## Abstract

Asymptomatic esophageal eosinophilia (aEE) is a rare presentation, where patients have increased eosinophils in esophageal mucosa but lack any esophagus-related symptoms. Cases of aEE have only been documented in adults, and little is known about its clinical significance and whether treatment is warranted. We report a case of an 11-year-old patient with uncontrolled severe persistent asthma who underwent flexible bronchoscopy and upper endoscopy as a part of complete aerodigestive evaluation. Elevated intraepithelial eosinophils in the esophageal mucosa were noted, suggesting an aEE-like presentation. This case documents a pediatric patient with aEE and highlights the importance of combined aerodigestive assessment with pulmonology and gastroenterology teams for the evaluation of severe asthma.

## 1. Introduction

Atopic diseases, such as atopic dermatitis and allergic asthma, are increasing in prevalence worldwide [1–3]. These atopic conditions are more commonly identified in patients with eosinophilic esophagitis (EoE), an atopic condition of the esophagus [4]. EoE is characterized by clinical features (e.g., dysphagia, food impaction, and upper abdominal pain) and histologic features (e.g., an esophageal biopsy with at least 15 eosinophils per high-powered microscopy field (hpf)) [5]. However, patients may undergo esophageal biopsy for a nonesophageal complaint, revealing elevated eosinophils in the esophagus without the associated clinical symptoms of EoE. Asymptomatic esophageal eosinophilia (aEE) is a rare condition where patients are found to have histologic features of EoE but lack the associated esophageal symptoms [6]. While the prevalence of EoE is known and rising, as patients need to be symptomatic to satisfy diagnostic criteria, cases of aEE are likely underreported due to the asymptomatic nature of the condition [7]. Furthermore, the significance of this clinical finding, and whether or not treatment is warranted, is unclear.

In the pediatric patient population, EoE is increasing in prevalence alongside atopic disease as a whole [1–3, 7, 8]. However, aEE has not yet been widely described in the pediatric patient population, as children without esophageal symptoms often lack clinical indication for upper gastrointestinal endoscopy and esophageal biopsy. In addition, there are few studies investigating a potential link between aEE and other clinical phenotypes, such as atopic disease. Here, we present the case of an eleven-year-old patient with severe persistent asthma but no baseline symptoms to suggest a concern for esophagitis, who underwent upper gastrointestinal endoscopy. Biopsies revealed elevated eosinophils in the esophageal mucosa, without associated esophageal symptoms.

The long-term complications of untreated EoE are clear: persistent allergic inflammation leading to fibrosis and esophageal strictures over time, resulting in permanent damage to the esophagus [9]. However, the long-term complications of aEE are more uncertain, bringing into question the importance to treat aEE, especially in the pediatric population where an emphasis on preventing long-term complications is necessary.

## 2. Case Presentation

An 11-year-old male patient with uncontrolled severe persistent asthma and allergic rhinitis underwent an upper gastrointestinal endoscopy and flexible bronchoscopy. A diagnosis of asthma was given six years prior following repeated episodes of cough, chest tightness, shortness of breath, and wheezing triggered by viral respiratory infections and exercise. Sneezing and coughing following exposure to environmental allergens was also noted. At this time, patient was already on Step 5 inhaled corticosteroid-long acting beta agonist (ICS-LABA) therapy montelukast, and as needed short acting beta agonist for rescue treatment [10]. Response to initial asthma treatment over the duration of care was limited, indicating a need for further management. In addition, since the initial asthma diagnosis, this patient was diagnosed with essential hypertension and hyperlipidemia in the context of obesity (patient's weight is >97th percentile). Amlodipine was prescribed; however, the patient was noncompliant.

Concurrently, this patient was also being followed by gastroenterology for functional constipation, where initial efforts to treat with polyethylene glycol were unsuccessful. The patient reported abdominal discomfort and ultimately discontinued its use without resolution of constipation. Coordination between gastroenterology and pulmonology teams concluded that a bronchoscopy and an upper endoscopy were required for further evaluation to rule out other factors in the pathogenesis of respiratory symptoms.

Bronchoscopy revealed that the airway mucosa had evidence of inflammation and friability, consistent with uncontrolled severe persistent asthma. Bronchoalveolar lavage (BAL) was performed in the lingula and right middle lobe, and upon gross observation, the BAL specimen was clear; however, microscopy revealed the presence of hemosiderin-laden macrophages, indicating bleeding into the airway (483WBC/mcL BAL fluid, 740RBC/mcL BAL fluid). Upper endoscopy revealed esophageal mucosal changes in the mid and distal esophagus, including linear erosions without hemorrhage, often seen with esophagitis. Four biopsies were obtained from the middle esophagus and distal esophagus. Microscopic analysis revealed 35 eosinophils per HPF in both areas with microabcesses and degranulation, confirming the histopathologic criterion for a diagnosis of eosinophilic esophagitis.

## 3. Discussion

GERD and asthma are often found as coexisting conditions, and GERD is recognized as a trigger in many cases of severe asthma [11]. However, it is controversial whether treatment of GERD and/or GERD symptoms improves asthma control. Hence, there is lack of consensus regarding the extent of gastrointestinal evaluation in patients with severe asthma especially those who deny any symptoms of GERD. Asymptomatic esophageal eosinophilia (aEE) is a rare presentation of elevated eosinophil infiltration (>15 eosinophils per high-powered field) within the esophageal mucosa in the absence of clinical esophageal symptoms [6]. This condition is documented in adult patients, often found incidentally during upper endoscopy for gastric cancer screening. Eosinophilic esophagitis (EoE) is a similar condition with elevated eosinophils in esophageal mucosa but is associated with esophageal symptoms to help satisfy diagnostic criteria [5]. These esophageal symptoms include dysphagia, food impaction, vomiting, abdominal pain, and chest pain. In severe cases, strictures can develop. Treatment of EoE can involve elimination diets, acid suppression with proton-pump inhibitors, and inflammation reduction with glucocorticoids [5]. For patients with severe EoE disease, biologics can now be used, but only after other treatment options are exhausted [12]. Importantly, the long-term goal of treatment is to limit the progression of esophageal inflammation to fibrosis and stenosis [9].

It is unclear whether the persistent, subclinical inflammation of aEE has similar long-term adverse effects. Few studies have aimed to investigate the molecular differences between eosinophilic inflammation in aEE compared to EoE. Using immune markers of eosinophilic inflammation (e.g., major basic protein, eosinophil-derived neurotoxin, and eotaxin-3), a study performed by Kitamura et al. identified no significant differences between aEE and EoE at the cellular level, supporting the hypothesis that aEE and EoE have similar pathophysiologic processes [7]. Importantly, for the pediatric population, long-term complications are of primary concern, as they can be difficult to manage and may lead to lifelong challenges.

This case documents aEE in a pediatric patient in the context of severe persistent asthma, which raises interesting questions about the etiology of aEE in the pediatric population. Although our patient lacked esophageal symptoms, the number of eosinophils per high-powered field (>35) was consistent with a diagnosis of EoE [5]. Furthermore, this patient had mild peripheral eosinophilia (serum eosinophil = 500 eosinophil/mcL (5.3%) on CBC), suggesting an atopic-like clinical picture. However, elevated circulating IgE and other classic atopic conditions were not present. Biologic therapies, such as omalizumab (anti-IgE) [13], mepolizumab (anti-IL-5) [14], and dupilumab (anti-IL-4Ra) [15, 16] have all been FDA-approved for use in children aged 6 years and older with moderate-to-severe asthma. However, dupilumab has also been approved for treatment of EoE in adults. Dupilumab is a monoclonal antibody that targets the IL-4 receptor alpha (IL-4Ra), a subunit for both IL-4 and IL-13 receptors that is present on many different immune cells, as well as respiratory epithelial and smooth muscle cells [17, 18]. Activation of these receptors promotes the allergic inflammation seen in both allergic asthma and EoE. For patients with severe persistent asthma and EoE (or aEE, as in the case presented here), dupilumab will be beneficial as it targets the same underlying pathogenic pathway.

The co-occurrence of aEE with severe persistent asthma in this 11-year-old patient brings to question several aspects of clinical management for consideration: (1) what is the significance of aEE in the pediatric population; (2) is treatment for aEE warranted; and (3) should upper gastrointestinal endoscopy be incorporated into routine clinical workup for pediatric patients with severe asthma even in the absence of esophageal symptoms. We suspect many patients with severe allergic asthma have some level of aEE that may warrant treatment and may need to be considered for a full evaluation by pulmonary and gastroenterology teams, despite the absence of symptoms for traditional EoE.
